# Effects of Exercise Modes on Neural Processing of Working Memory in Late Middle-Aged Adults: An fMRI Study

**DOI:** 10.3389/fnagi.2019.00224

**Published:** 2019-09-04

**Authors:** Feng-Tzu Chen, Ya-Ping Chen, Stefan Schneider, Shih-Chun Kao, Chih-Mao Huang, Yu-Kai Chang

**Affiliations:** ^1^Department of Physical Education, National Taiwan Normal University, Taipei, Taiwan; ^2^Centre for Cognitive Neuroscience, University of Salzburg, Salzburg, Austria; ^3^Institute of Movement and Neurosciences, German Sport University Cologne, Cologne, Germany; ^4^Department of Health and Kinesiology, Purdue University, West Lafayette, IN, United States; ^5^Department of Biological Science and Technology, National Chiao Tung University, Hsinchu, Taiwan; ^6^Cognitive Neuroscience Laboratory, Institute of Linguistics, Academia Sinica, Taipei, Taiwan; ^7^Institute for Research Excellence in Learning Science, National Taiwan Normal University, Taipei, Taiwan

**Keywords:** open-skill exercise, closed-skill exercise, executive control, aging, cognition

## Abstract

Recent studies have highlighted the importance of regular exercise on cognitive function in aging populations, with aerobic exercise and cardiovascular fitness having received the largest amount of research attention. However, the relationship between exercise mode and cognitive function underlying behavioral modification and neural activation remains unknown. The present study, therefore, sought to examine the associations between different exercise modes and the working memory (WM) aspect of executive function as well as its task-evoked brain activation in the late middle-aged population. Seventy late middle-aged adults were classified into open-skill, closed-skill, or irregular exercise groups based on their participation in exercise activities prior to the study and then performed a spatial working memory (SWM) task while undergoing functional magnetic resonance imaging (fMRI) scanning. The results revealed that exercise groups, regardless of exercise modes, showed better SWM and physical fitness performance. Additionally, the open-skill group exhibited greater brain activation in the prefrontal lobe, anterior cingulate cortex/supplementary motor area (ACC/SMA), and hippocampus than those in the closed-skill group, suggesting a mode-sensitive compensatory mechanism in late middle-aged adults. These findings indicate that exercise promotes cognitive health, improves WM, and enhances neurocognitive scaffolding in late middle-aged adults and further suggest that various exercise modes can effectively modulate frontal and hippocampal function in the face of age-related neurocognitive declines, implications that may inform the development of exercise programs for the elderly.

## Introduction

Increasing age is characterized by a progressive decline in many aspects of cognitive function, such as processing speed, working memory (WM), long-term memory, and reasoning (Park and Reuter-Lorenz, 2009; Salthouse, 2010). These cognitive declines are associated with increased risk of mild cognitive impairment (MCI) or dementia (Petersen et al., 2010), as well as losses in structural and functional brain (Hedden and Gabrieli, 2004), which are linked with neuropsychiatric symptoms and disability. These age-related cognitive impairments are of particular public health concern due to the personal and economic burdens they bring (Andersen et al., 2004), and it is therefore urgent to identify cost-effective strategies for mitigating their impacts in aging populations.

Exercise has been linked to enhanced cognitive functioning and reduced risk of cognitive impairment in animals (García-Mesa et al., 2011) and older humans (Netz et al., 2011; Behrman and Ebmeier, 2014; Frederiksen et al., 2015; Miller and Taylor-Piliae, 2018). The effects of exercise on cognitive function appear to be particularly beneficial to executive control. More specifically, compared with other cognitive domains (e.g., those relating to processing speed and spatial perceptions), executive control is particularly affected by long-term exercise interventions in older adults (Colcombe and Kramer, 2003). Executive control, also known as cognitive control or executive function, refers to a high-order cognitive process that involves three different subcomponents, namely, the inhibition of proponent responses, the shifting of mental sets, and the updating of WM (Miyake et al., 2000). Previous studies have thoroughly documented that cardiovascular fitness is positively associated with the subcomponents of inhibition and shifting (Themanson et al., 2006; Gothe et al., 2013; Chang et al., 2014; Fong et al., 2014; Leckie et al., 2014), as well as WM (Guiney and Machado, 2013).

Of the three subcomponents, WM is the least investigated in terms of its relationship with exercise and executive function. WM refers to the cognitive process through which complex information is retained and manipulated over a short period of time, and it is essential to the integration of information (Fuster and Bressler, 2012) and the optimal performance of goal-oriented behaviors (Gazzaley and Nobre, 2012). The distributed network of WM involves a group of frontal and parietal lobes, including the inferior frontal gyrus (IFG), anterior cingulate gyrus/supplementary motor area (ACC/SMA), hippocampus, and thalamus (Herrero et al., 2002; Yin et al., 2013; Gutiérrez-Garralda et al., 2014; Toepper et al., 2014). Furthermore, WM is particularly susceptible to precipitous declines with age (Park and Reuter-Lorenz, 2009; Schmiedek et al., 2009), and impairments in WM are associated with behavior disorders and social difficulties (Baddeley, 1992; Engle et al., 1999; Dennis et al., 2009), and contribute to decreased quality of life and reduced functional independence in aging populations.

The positive relationship between cardiovascular fitness and brain function has been supported by various studies employing functional magnetic resonance imaging (fMRI). For instance, Colcombe et al. (2004) observed that high-fit (in terms of cardiovascular fitness) older adults exhibited higher task-related activation in the frontal and parietal cortical regions (e.g., the middle frontal gyrus, superior frontal gyrus, and superior and interior parietal lobules) and lower activation in the anterior cingulate regions (e.g., ACC) when performing a task (the Flanker task) involving inhibition than low-fit older adults. More recently, Prehn et al. (2019) observed increased activation during a task involving shifting in the prefrontal lobes and superior parietal gyrus/precuneus in subjects who participated in a 6-months aerobic training program compared to those who took part in a 6-months stretching and toning program. Taken together, these studies suggested that cardiovascular fitness is associated with executive control and its associated brain functions in the frontal, parietal, and temporal regions required for the inhibition and shifting aspects of executive control. Notably, the activation of these regions is also associated with WM, suggesting that the beneficial effects of cardiovascular fitness could extend to WM.

Aside from aerobic exercise that primarily induces cardiovascular fitness, a systematic review, showed that training programs combining multiple forms of exercise are also beneficial to cognitive function in older adults with and without cognitive impairment (Law et al., 2014). Furthermore, along with improved cognitive performance, increased brain function has also been observed following various types of exercise training. For example, Voelcker-Rehage et al. (2010) indicated that older adults with higher motor fitness (e.g., movement speed, balance, fine coordination, and flexibility) exhibited differential brain activation related to control processes compared to those with higher physical fitness (i.e., cardiovascular fitness and muscular strength). Thus, it is possible that exercise modes associated with different forms of fitness may activate brain patterns in distinct manners.

Different forms of exercise can be classified into two major types: open-skill exercises (e.g., tennis, table tennis, badminton) and closed-skill exercises (e.g., jogging, cycling, swimming; Schmidt and Wrisberg, 2008). Open-skill exercises aim to improve individual strength, agility, flexibility, and power, and entail higher cognitive demands in order to immediatedly repond to external stimuli in an unpredictable environment, whereas closed-skill exercises aim to improve cardiovascular fitness through the repetition of routine movements and actions. Using the event-related potential (ERP) approach, Huang et al. (2014) found that subjects who regularly engaged in open-skill exercises exhibited larger P300 amplitudes during the required inhibition aspect of executive control when performing the Flanker task than those who regularly engaged in closed-skill exercises or those who only exercised on an irregular basis, suggesting that open-skill exericise leads to the allocation of greater attentional resources and increased response efficiency compared with closed-skill exercise or irregular exercise. However, Dai et al. (2013) reported that subjects who did either open-skill or closed-skill exercise, regardless of exercise mode, exhibited shorter response times (RTs) and larger P300 amplitudes on a task-switching task. Similar results were also reported by Tsai and Wang (2015), who found that subjects included in either of two types of regular exercise groups exhibited faster RTs and larger P200 and P300 amplitudes than those who engaged in irregular exercise. These inconsistent results suggest that the effects of different exercise modes on executive function require further examintion, not to mention the fact that previous studies of open-skill vs. closed-skill exercise have yet to focus on WM. Moreover, the evidence from the aforementioned studies was limited to behavioral and ERP-related results, whereas the differential effects of different exercise modes on brain functioning have yet to be investigated using fMRI.

Exercises that entail significant cognitive demands, such as open-skill exercises, may alter cognitive functioning and result in brain activation changes that are associated with WM. For example, previous studies have suggested that relative to closed skills, open skills may exhibit similar and additional facilitating effects on the cognition of older adults (Tranter and Koutstaal, 2008; Taddei et al., 2012). Additionally, a longitutinal fMRI study conducted by Nishiguchi et al. (2015) indicated that older adults participating in a randomized control trial of a 12-weeks combined cognitive and physical exercise training program (i.e., one involving dual-task exercises) exhibited less activiation in the prefrontal cortex related to WM than those placed on a waiting list, suggesting that exercise training entailing significant cognitive demands can affect the efficiency of brain functioning during the performance of WM-related tasks in older adults.

Accordingly, higher cardiovascular fitness and higher rates of participation in exercise have been linked to better executive control, in terms of inhibition and shifting, in older adults. However, few studies have examined the effects of different exercise modes on WM and its neural correlates in late middle-aged adults. Therefore, the present study sought to examine whether and how different exercise modes impact the neural correlates of WM processing by utilizing fMRI. We hypothesized that open-skill exercise and closed-skill exercise would elicit better WM performance and induce greater activation in the fronto-parietal regions of the middle-aged brain than irregular exercise. In addition, we expected that the participants with open-skill exercise experience would show differential patterns of brain activation in the subcortical regions compared to those with closed-skill exercise experience.

## Materials and Methods

### Participants

Seventy community-dwelling middle-aged adults (mean age = 58.40 years; females *n* = 30) were recruited from several sport and exercise centers located in two districts and at three universities in Taipei *via* public advertisements (e.g., flyers, website information). The inclusion criteria for this study required that the patients have: (1) right-hand dominance; (2) normal or corrected-to-normal vision; (3) no neurological disorders; (4) no diagnosis of psychiatric disorders; (5) no use of psychoactive medication; and (6) a Mini-Mental State Examination (MMSE) score of more than 25. The participants were then allocated into one of three groups, an open-skill exercise group, closed-skill exercise group, or irregular exercise group, based upon their prior participation in different forms of exercise. Specifically, the participants in the open-skill exercise group and closed-skill exercise group had to have participated in, respectively, open-skill exercises (i.e., basketball, tennis, table tennis, and badminton) or closed-skill exercises (i.e., jogging, cycling, and swimming) with moderate intensity at least three times per week for 30 min per session for over 3 months. These exercise experiences were assessed by a self-report questionnaire, which consisted of items indicating: (1) the number of years that the participant had regularly participated in the specific exercise mode; (2) the exercise duration per session; and (3) the exercise frequency per week. The participants also filled out the International Physical Activity Questionnaire (IPAQ; Liou et al., 2008) to indicate their amount of physical activity over the past 7 days. The participants in the irregular exercise group, meanwhile, consisted of those who had engaged only in irregular exercise or low-intensity exercise within the past 3 months. The individual sub-scale intelligence of the eligible participants was also evaluated by the administration of the digit span test of the *Wechsler* *Adult Intelligence Scale—Third Edition* (Wechsler and Corporation, 1997).

All of the participants received verbal and written explanations of the study and voluntarily completed the written informed consent, and the study received ethical approval from the Institutional Review Board of National Taiwan University.

### Physical Fitness Assessment

A general physical fitness test was administered to assess six indices of health- and skill-related physical fitness (Chang et al., 2017; Song et al., 2017). Health-related fitness included cardiovascular fitness, muscular strength, muscular endurance, flexibility, and body composition; and skill-related fitness included agility and power.

The cardiovascular fitness was assessed by the Kasch step test (Golding, 2000), which is a relatively quick and convenient test for examining VO_2peak_ that is also referred to as the YMCA 3-min step test. The test requires the test taker to repeatedly step up on a bench and back down to the floor to the clicking of a metronome for 3 min. Muscular strength was assessed by a hand-grip test according to the average value for each hand. Muscular endurance was determined by 60-s push-up as well as 30-s and 60-s abdominal sit-ups. Flexibility related to the lower back and hamstrings was measured using the sit-and-reach test. Body mass index (BMI) was calculated using the formula weight (kg)/height^2^ (m). Agility was assessed using the *T*-test, in which the given participant was required to run as quickly as possible around four cones arranged in a *T* shape, and power was assessed using a vertical jump test that required the participant to jump as high as possible within a circular area.

### Working Memory fMRI Task

A computerized version of an event-related design of the spatial working memory (SWM) task (Sternberg, 1966) was programmed using E-prime (Psychology Software Tools, Pittsburgh, PA, USA). The SWM task included three WM load conditions (e.g., 1 dot, 3 dots, and 7 dots), and a total of 46 trials combining the three conditions were presented in each of two experimental sessions. During each trial, the given participant was required to react to the presentation of a white-colored stimulus by pressing the left or right button on a keyboard to indicate whether the probe display showed a dot in one of the identical locations (match condition) of the dot or dots shown in the encoding display or in a different location (non-match condition). After the probe periods, the following trials were jittered and randomly separated by inter-stimulus intervals of 10.5 s, 13.5 s or 15.5 s with the background being blank during those intervals. The RT and accuracy were recorded as the index of WM performance.

**Figure 1:**
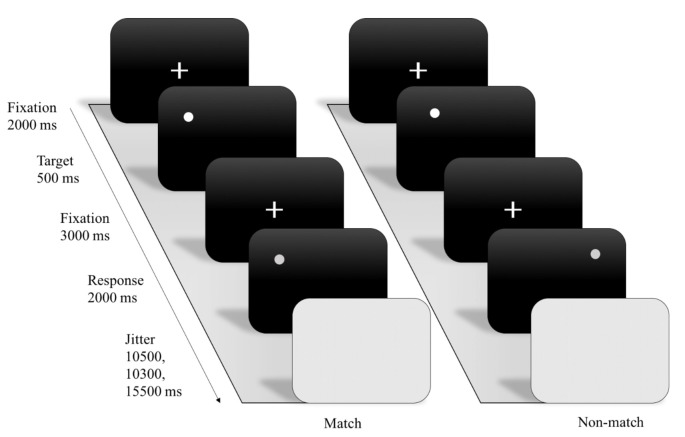
Example of experimental stimuli and procedure for spatial working memory (SWM) functional magnetic resonance imaging (fMRI) paradigm.

Through examining SWM task, we assess the blood oxygenation level-dependent (BOLD) response during the encoding phase and the timing was adapted for fMRI scanning. For each trial, the SWM paradigm included a fixation (2,000 ms) with white color presented on a black screen, followed by an encoding (500 ms) display with one of three working-memory load conditions. After 3,000 ms, a delayed fixation with white color was presented on a gray screen. Next, a 2,000-ms probe display was presented with a single dot that appeared either in a location previously shown (match) or not shown (non-match; see Figure 1).

Prior to starting the experiment, each participant performed a practice session of the SWM task consisting of a total of 10 trials with the three conditions. Each participant was instructed on how to respond correctly to each stimulus, and was allowed to ask questions regarding the task. Each participant was required to achieve more than 70% accuracy in the practice session prior to starting the official experiment. The accuracy and RT data were employed as the analyzed data.

### MRI Data Acquisition and Preprocessing

All the brain images were captured using a 3.0 T MR system (Siemens, Magnetom Skyra, Germany) with a 32-channel head coil array at the Taiwan Mind and Brain Imaging Center of National Chengchi University, Taipei. During the fMRI acquisition, the images were acquired using an echo-planar imaging (EPI) sequence (TR/TE/flip angle = 2,000 ms/30 ms/90 degree). Thirty-two contiguous axial slices were acquired with a slice thickness of 3.0 mm, 220 × 220 mm field of view (FOV), 64 × 64 matrix size, and 3.4 × 3.4 mm in-plane resolution. High resolution three-dimensional magnetization-prepared rapid gradient-echo (MPRAGE) structural images (voxel size = 0.5 × 0.5 × 7.0 mm^3^) were collected for spatial mapping and registration of the fMRI data. During the entire fMRI session, each participant was required to place padding carefully under his or her knees, between his or her head and the coil, and under his or her right forearm in order to minimize discomfort, minimize muscle strain, and physically minimize head motion during the scanning.

All preprocessing and general linear model (GLM) estimation was carried out using SPM8 software (Wellcome Department of Cognitive Neurology, Institute of Neurology, London, UK). The functional images were initially corrected for slice acquisition time and for head motion, followed by spatial normalization to the Montreal Neurological Institute (MNI) template and spatial smoothing using an 8-mm full-width and half maximum (FWHM) Gaussian kernel.

### fMRI Data Analysis

After preprocessing, the event-related BOLD responses for each experimental condition were separately modeled for each participant by convolving a vector of the onset times of the stimuli with the canonical hemodynamic response function (HRF) with the task paradigm within the context of the GLM implemented in SPM 8. To identify the brain areas associated with SWM, we contrasted the task conditions (i.e., the 1-dot, 3-dot, and 7-dot conditions) for each participant. Parameter estimates from the resulting contrast maps were then subjected to a second-level random-effects analysis to identify the brain regions that were significantly activated according to the contrast across participants. A voxel-wise analysis of variance (ANOVA) was conducted to detect the activated voxels associated with the task conditions across the three exercise mode groups. Regions of activated clusters were considered significant using a cluster-level threshold of *p* < 0.05 after family-wise error (FWE) corrections with a minimum cluster size of 10 contiguous voxels.

## Results

### Participants’ Characteristics and Exercise Experience

The participants’ characteristics including demographics and exercise experience are reported in Table 1. A one-way ANOVA performed on the participants’ characteristics and physical fitness indices revealed no significant difference in the majority of demographics among the three groups (*F*_(2,67)_ = 0.26–1.01, *p*s > 0.05). Regarding exercise experience, the one-way ANOVA revealed a significant difference in IPAQ (*F*_(2,67)_ = 8.30, *p* < 0.001) results among the three groups. *Post hoc* analysis showed that the closed-skill exercise group had the highest mean IPAQ score, followed by the open-skill exercise group and then the irregular exercise group. Additional exercise information including the number of years, duration per session, and frequency per week of exercise are also shown in Table 1.

**Table 1:** Participant characteristics and physical fitness indices across the three groups (mean ± standard deviation).

	Groups		
	Open-skill	Closed-skill	Irreg. exercise
**Demographics**			
Total/Female (%)	23/4 (17%)	24/15 (63%)	23/11 (48%)
Age (years)	57.17 ± 3.23	59.08 ± 7.15	58.91 ± 4.77
Weight (kg)	71.48 ± 10.68^a^	63.00 ± 10.53	64.17 ± 10.58
BMI (kg/m^2^)	24.59 ± 3.11	24.20 ± 2.77	24.33 ± 3.02
Education (years)	13.74 ± 2.03	12.46 ± 3.75	12.48 ± 4.34
MMSE	28.09 ± 1.62	27.54 ± 2.15	27.00 ± 3.00
Digit Span	20.09 ± 3.77	18.71 ± 4.47	19.91 ± 4.23
**Exercise experience**			
Years (regular)	15.20 ± 10.19	8.79 ± 5.14	N/A
Duration/session (min)	99.13 ± 30.59	79.17 ± 43.63	N/A
Frequency/week	3.26 ± 1.36	5.29 ± 1.52	N/A
IPAQ	1,476.59 ± 981.00^a^	1,869.42 ± 1,033.93^a, c^	420.95 ± 439.29
**Physical fitness**			
Cardiovascular fitness	72.38 ± 11.75^a^	70.80 ± 9.28^a^	60.53 ± 6.26
Muscular strength			
Right hand	42.87 ± 12.85^a,b^	33.06 ± 10.40	35.48 ± 10.29
Left hand	41.77 ± 7.36^a,b^	31.17 ± 8.94	33.67 ± 9.62
Muscular endurance			
Push-up	13.91 ± 10.21^a,b^	7.35 ± 5.26	4.39 ± 4.97
ASU 30	16.14 ± 5.38^a,b^	9.39 ± 6.53	7.30 ± 4.90
ASU 60	26.73 ± 9.36^a,b^	15.96 ± 11.93	12.26 ± 8.44
Flexibility (cm)	15.64 ± 11.55	25.13 ± 10.46^c^	21.57 ± 11.56
Agility (ms)	12.18 ± 1.59^a,b^	16.68 ± 3.18	18.22 ± 3.18
Power (cm)	45.86 ± 12.80^a,b^	28.87 ± 9.32	30.76 ± 11.25

*Notes: BMI, Body mass index; MMSE, Mini-Mental State Examination; IPAQ, International Physical Activity Questionnaire; ASU 30, abdominal sit-ups of 30 s; ASU 60, abdominal sit-ups of 60 s; ^a^Indicates significant difference compared to the irregular exercise group, *p* < 0.05; ^b^indicates significant difference compared to the closed-skill exercise group, *p* < 0.05; ^c^indicates significant difference compared to the open-skill exercise group, *p* < 0.05*.

### Physical Fitness Results

The participants’ physical fitness data are reported in Table 1. A one-way ANOVA revealed that there were significant differences in all the indices of physical fitness (*F*_(2,67)_ = 4.11–28.77, *p*s = 0.00–0.02). A *post hoc* analysis of the cardiovascular fitness results revealed that the open-skill exercise and closed-skill exercise groups had greater cardiovascular fitness than the irregular exercise group (*p*s < 0.01). Moreover, *post hoc* analyses revealed that the open-skill exercise group had better performance than both the closed-skill exercise group and the irregular exercise group in terms of muscular strength/right hand (*p*s < 0.01), muscular strength/left hand (*p*s < 0.01), push-up (*p*s < 0.01), 30-s abdominal sit-up (*p*s < 0.01), 60-s abdominal sit-up (*p*s < 0.01), agility (*p*s < 0.01), and power (*p*s < 0.01). An analysis of the flexibility results revealed that the closed-skill exercise group was more flexible than the open-skill exercise group (*p* < 0.05) and had an equivalent performance with irregular exercise group (*p* = 0.28).

### Working Memory Behavioral Results

**Figure 2:**
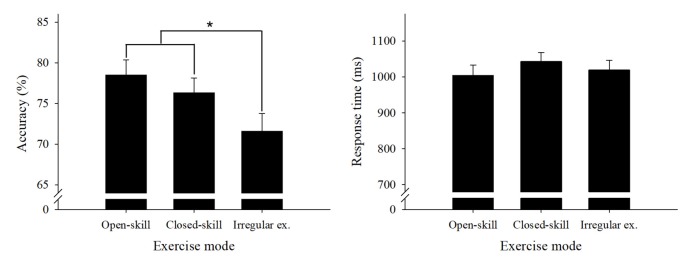
Global accuracy and response time (RT) behavioral performance results (mean and standard error) for the spatial working memory (WM) task across the three groups. Open-skill, open-skill exercise group; closed-skill, closed-skill exercise group; Irregular ex., irregular exercise group. *Indicates significant difference between groups, *p* < 0.05.

Table 2 presents the behavioral results. We performed a two way 3 × 3 ANOVA on the accuracy data with group (open-skill exercise, closed-skill exercise, and irregular exercise) as a between-subject factor and condition (1-dot condition, 3-dot condition, and 7-dot condition) as a within-subject factor. The results revealed significant main effects of *group* (*F*_(2,67)_ = 5.73, *P* < 0.01, partial *η*^2^ = 0.15) and *condition* (*F*_(2,134)_ = 318.65, *p* = 0.00, partial *η*^2^ = 0.83), but no *interaction* between group and task condition (*p* > 0.05). Multiple follow-up comparisons for the *group* factor revealed that the open-skill and closed-skill exercise groups had significantly higher accuracy, with no significant difference between them, than the irregular exercise group (*p*s < 0.05, Figure 2). Multiple follow-up comparisons for the *task condition* revealed that accuracy was significantly higher in the 1-dot condition than the 3-dot and 7-dot conditions (*p*s < 0.05), while the 3-dot condition had significantly higher accuracy than the 7-dot condition (*p* < 0.05).

**Table 2:** Behavioral performance in spatial working memory task across the three groups.

	Group		
Conditions	Open-skill	Closed-skill	Irreg. exercise
**Accuracy (%)**			
1-dot	94.87 ± 4.92*	91.33 ± 8.29*	89.82 ± 10.35
3-dot	78.65 ± 7.87*	76.54 ± 11.96*	71.39 ± 13.20
7-dot	62.00 ± 9.51*	61.04 ± 8.11*	53.52 ± 8.91
**Response time (ms)**			
1-dot	821.39 ± 36.05	850.36 ± 32.01	844.51 ± 35.87
3-dot	1,033.79 ± 44.24	1,090.78 ± 34.54	1,038.56 ± 35.59
7-dot	1,156.80 ± 44.07	1,188.16 ± 30.86	1,174.11 ± 43.09

Note: *indicates significant difference compared to the irregular exercise group, *p* < 0.05.

Performance of the same group × task condition ANOVA on the RT data (Figure 2) revealed a significant main effect for *task condition* (*F*_(2,134)_ = 376.00, *p* = 0.00, partial *η*^2^ = 0.85) but not for *group* (*F*_(2,67)_ = 0.69, *p* > 0.05, partial *η*^2^ = 0.02), as well as an *interaction* *effect* between task condition and group (*p* > 0.05). A follow-up comparison for *task* *condition* showed a significantly slower RT in the 7-dot condition as compared with the 3-dot condition and the 1-dot condition (*p*s < 0.05), while the 3-dot condition had a significantly slower RT than the 1-dot condition (*p* < 0.05).

### fMRI Results on Working Memory

**Figure 3:**
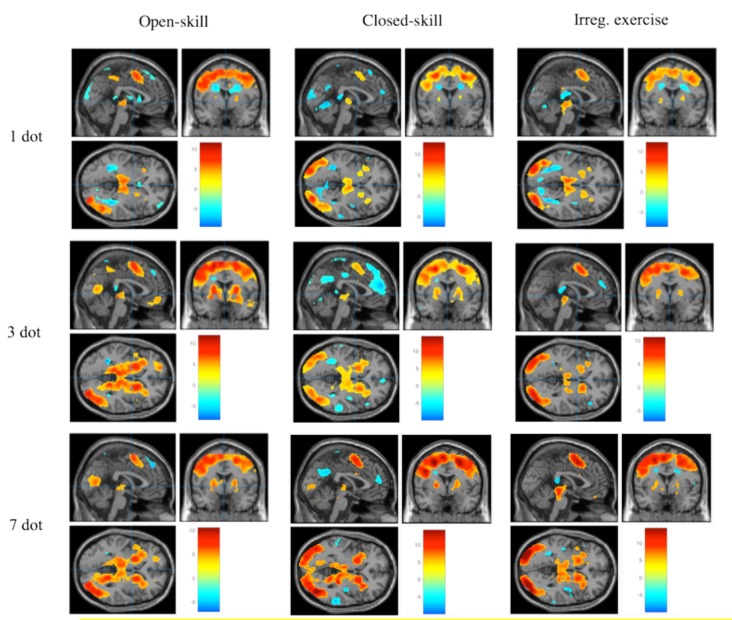
Brain activation during spatial WM task for each group and each condition (≧ 10 contiguous voxels with FWE *p* < 0.05 correction).

Figure 3 demonstrates the significant brain activation associated with the WM task in the different task conditions across the three exercise mode groups. The detailed information of the brain regions is shown in Tables s 3, 4. The ANOVA results revealed significant group differences in the frontal cortex, subcortical regions, and hippocampus. Specifically, the open-skill exercise group showed significantly greater activation than the closed-skill exercise group in the left IFG, left thalamus, right hippocampus, left putamen, and left anterior ACC/SMA. There was no significant difference between the closed-skill exercise group and the irregular exercise group; however, the closed-skill group showed significantly reduced activation in the right hippocampus compared to the irregular exercise group (3).

**Table 3:** Brain regions showing group differences in neural activation during spatial working memory task.

	*x*	*y*	*z*	Open-skill	Closed-skill	Irreg. exercise
**Left sphere**						
IFG	−32	22	4	4.33 ± 2.29^b^	3.35 ± 3.49	3.49 ± 2.52
ACC/SMA	−8	8	48	2.79 ± 1.95^b^	1.97 ± 1.29	2.68 ± 2.04
Thalamus	−6	26	−4	3.28 ± 2.46^b^	1.82 ± 2.19	2.42 ± 2.10
Putamen	−20	10	4	2.88 ± 2.62^b^	1.74 ± 1.83	2.17 ± 2.33
DLPF	−34	−4	48	3.47 ± 2.07	3.23 ± 1.88	3.39 ± 2.21
**Right sphere**						
Hippocampus	8	−26	−6	3.40 ± 2.46^a,b^	1.62 ± 1.83^a^	2.53 ± 1.97^b^
IFG	32	22	6	1.29 ± 1.64	1.16 ± 1.19	1.17 ± 1.65
Thalamus	14	−22	6	1.68 ± 1.66	1.25 ± 1.52	1.37 ± 1.92
Putamen	20	8	0	2.91 ± 2.47	2.07 ± 1.87	2.48 ± 2.38

*Notes: IFG, inferior frontal gyrus; ACC/SMA, anterior cingulate cortex/supplementary motor area; DLPF, dorsolateral prefrontal; ^a^indicates significant difference compared to the irregular exercise group, *p* < 0.05; ^b^indicates significant difference compared to the closed-skill exercise group, *p* < 0.05*.

**Table 4:** Brain regions showing condition-related differences in neural activation during spatial working memory task.

	*x*	*y*	*z*	1-dot	3-dot	7-dot
**Left sphere**						
IFG	−32	22	4	2.81 ± 2.03	4.04 ± 2.12^a^	4.30 ± 2.56^a^
ACC/SMA	−8	8	48	2.05 ± 1.57	2.67 ± 1.83^a^	2.71 ± 1.96^a^
Thalamus	−6	26	−4	2.10 ± 2.16	2.54 ± 2.20	2.85 ± 2.55^a,b^
Putamen	−20	10	4	1.30 ± 1.97	2.64 ± 2.12^a^	2.88 ± 2.52^a^
DLPF	−34	−4	48	2.63 ± 1.81	3.67 ± 2.17^a^	3.87 ± 2.43^a^
**Right sphere**						
Hippocampus	8	−26	−6	2.11 ± 2.08	2.46 ± 2.15	2.94 ± 2.35^a,b^
IFG	32	22	6	0.82 ± 1.40	1.17 ± 1.44^a^	1.64 ± 1.56^a,b^
Thalamus	14	−22	6	1.23 ± 1.59	1.46 ± 1.80	1.60 ± 1.72
Putamen	20	8	0	1.58 ± 2.04	2.85 ± 2.06^a^	3.01 ± 2.42^a^

*Notes: IFG, inferior frontal gyrus; ACC/SMA, anterior cingulate cortex/supplementary motor area; DLPF, dorsolateral prefrontal; ^a^indicates significant difference compared to the 1-dot condition, *p* < 0.05; ^b^indicates significant difference compared to the 3-dot condition, *p* < 0.05*.

The ANOVA results revealed significant *task condition differences* in the frontal and subcortical regions. The 3-dot and 7-dot conditions showed greater activation in the bilateral IFG, left dorsolateral prefrontal cortex (DLPFC), left ACC/SMA, and bilateral basal ganglia compared with 1-dot condition. The 7-dot condition showed greater activation in the right IFG, right hippocampus, and left thalamus (Table 4). An *interaction* effect of group and task condition was observed in the putamen, with the open-skill exercise group demonstrating significantly increased activation for the 3-dot and 7-dot conditions, but not for the 1-dot condition, compared to the closed-skill exercise group.

## Discussion

The present task-related fMRI study examined the influences of open-skill and closed-skill exercise modes on physical fitness, cognitive performance, and neural activities associated with WM in late middle-aged adults. The behavioral results demonstrated a superior effect of open-skill exercise on physical fitness, with the open-skill exercise group exhibiting better performance than both the closed-skill exercise group and the irregular exercise group in terms of muscular strength/right hand, muscular strength/left hand, push-up, 30-s abdominal sit-up, 60-s abdominal sit-ups, agility, and power. Moreover, both the open-skill and closed-skill exercise groups showed better WM performance than those in the irregular exercise group, indicating the beneficial effects of exercise in terms of promoting cognitive health during aging. Moreover, the fMRI results demonstrated that the open-skill exercise group showed greater activation in the left IFG, left ACC/SMA, left thalamus, and right hippocampus during the WM task compared to those in the closed-skill exercise group, suggesting a mode-sensitive compensatory mechanism in middle-aged adults whose exercise activities involve more executive components in the WM domain. Our findings provide supportive behavioral and neuroimaginug evidence that various types of exercise modes improve human cognitive, phsyical, and brain functioning, and push forward our understanding that differerent exercise modes can modulate the neural processing of WM in the late middle-aged population.

The present study found that the late middle-aged adults in the open-skill and closed-skill exercise groups exhibited higher cardiovascular fitness and greater behavioral performance in terms of WM than those in the irregular exercise group. These findings are consistent with those of previous studies observing that older adult individuals with higher cardiovasuclar fitness exhibit greater behavioral accuracy in terms of WM (Oberlin et al., 2016). Similarly, high levels of cardiovascular fitness have been found to result in greater encoding and retrieval executive control processes of WM in child populations (Chaddock et al., 2011; Khan and Hillman, 2014). Our findings are also consistent with growing literature indicating a beneficial association between higher cardiovascular fitness and other aspects of executive control involving inhibition and switching (Scisco et al., 2008; Smiley-Oyen et al., 2008; Chapman et al., 2013). Our behavioral findings support the notion that cardiovascular fitness promotes cognitive health during aging and effectively improves WM in late middle-aged adults.

Our results indicated that various types of exercise mode influence cognitive function and physical fitness. Both the open-skill and closed-skill exericse groups showed similar positive effects in terms of WM performance compared to the irregular exercise group, which is in line with several studies showing that older adults participating in both open-skill and closed-skill exercise exhibit greater inhibition (Huang et al., 2014; Li et al., 2018) and switching (Dai et al., 2013) capabilities. Notably, the open-skills group in the present study also exhibited higher abilities in terms of a variety of fitness indices, including muscular strength, muscular endurance, agility, and power, compared to the other two groups. These findings suggest that open-skill exercise and closed-skill exercise may scaffold the physiological and/or neurocognitive mechanisms of the middle-aged brain in adaptive ways (Park and Reuter-Lorenz, 2009), which is congruent with the ERP-related implications of older adults participating in open-skill exercise showing larger P3 amplitudes than those engaging in closed-skill exercise when performing the switch condition of a task-switching task (Tsai and Wang, 2015).

In this study, significant group differences in the brain activation of the frontal cortex were also found between the open-skill and closed-skill exercise groups. Compared to the closed-skill group, the open-skill group exhibited significantly greater brain activation in response to the WM task across all the conditions in the left IFG and ACC/SMA. The prefrontal lobe acts to actively maintain and manipulate goal-directed representations, which are associated with the ability of executive control (Miller and Cohen, 2001). Given that the regions of the IFG and ACC/SMA have been suggested to be vulnerable to age-related structural and functional changes (Grady et al., 2006), our finding that the open-skill group exhibited increased activation in the prefrontal regions when utilizing WM suggests a compensatory mechanism for middle-aged adults who engage in exercise with open-skill features; that is, the brains of these adults appear to flexibly recruit more neural resources to compensate for losses in cognitive performance and neural efficiency due to age-related neurodegradation of the brain (Park and Reuter-Lorenz, 2009; Grady, 2012; Huang et al., 2012). Specifically, the IFG is the region for executive processing, which comprises the encoding and retrieval processing of short-term memory (Marklund and Persson, 2012). Previous neuroimaging studies have shown a positive association between high cardiovascular fitness and increased IFG volume in older adults (Weinstein et al., 2012; Voss et al., 2013). More importantly, the open-skill group, but not the closed-skill group, in this study showed greater IFG activation when utilizing WM, suggesting that exercise modes differentially contribute frontal-related compensatory processing in the late middle-aged population, a phenomenon that may inform the selection of targeted activities for future exercise programs for the elderly.

The ACC is thought to play an important role in monitoring processing, which is connected to executive control related to inhibition and switching ability (Fuentes-Claramonte et al., 2015; Duffy et al., 2016). The present study is the first to report that a closed-skill exercise group exhibited lower activation than an open-skills group in the ACC region. This finding was partially in line with one from a study by Colcombe et al. (2004), who found that older adults with high cardiovascular fitness exhibited decreased activation in the ACC compared to those with low cardiovascular fitness; however, we have expanded upon that knowledge by showing that exercise with open-skill features induced brain activity changes in the ACC/SMA during a WM task in late-middle-aged adults.

In addition to the prefrontal regions, the left thalamus also revealed greater activation for the open-skill group in this study compared with the closed-skill group. Relatedly, Hayes et al. (2017) demonstrated that healthy older adults with greater cardiovascular fitness showed increased subcortical activation in the thalamus than those with low cardiovascular fitness during a face-name WM task. Given that this face-name task is associated with the encoding and retrieval processes of relational information in WM, our fMRI results for the spatial WM task utilized in this study suggest a domain-general effect of cardiovascular fitness on the thalamus and WM in late middle-aged adults.

Interestingly, the different exercise modes modulated brain activation in the hippocampus during the WM task in different ways, with the open-skill group exhibiting greater hippocampal activation and the closed-skill group showing reduced hippocampal activation. The hippocampus is located within the medial temporal lobe that supports relational information, spatial cognition, and spatial navigation, which has been recognized as a critical brain region for allocating representations in cognitive maps (i.e., directions and distances between objects; Cohen et al., 1999; Parslow et al., 2004). Findings from animal research have suggested that exercise is associated with higher cerebral blood volume (CBV) in the dentate gyrus of the hippocampus (Pereira et al., 2007). In addition, individuals performing spatial WM tasks involving complex environments have shown different patterns of hippocampal activity during those tasks (Wolbers and Büchel, 2005). Given the notion that different types of fitness facilitate and improve physiological and brain function, our results suggest that distinct representations of cognitive maps involved in different exercise modes (i.e., open-skill vs. closed-skill exercise) could protect against age-related and/or disease-related declines in hippocampal function.

The present study is one of only a few studies to examine whether and how various types of exercise modes influence the neural functions of WM in late middle-aged adults using fMRI; thus, some limitations of this study should be acknowledged. First, given its cross-sectional design, the causal effects of the different exercise modes on WM remain unknown, so further studies employing longitudinal interventions are encouraged. Second, the present study only targeted spatial WM, so different types of WM (i.e., N-back; Kato et al., 2018) and even different cognitive domains should be explored in follow-up studies. Third, past studies have demonstrated that older adults engaged in cognitive or sensorimotor training utilize reduced brain resources (i.e., activation, glucose metabolic rate) relative to healthy populations (Degen and Schröder, 2014) and people with schizophrenia (Haier et al., 1992), which suggests that this kind of intervention facilitates brain efficiency. Therefore, we suggest that future studies should consider comparing the effects of different interventions on the utilization of brain resources. Finally, genetic variations, such as variations in the apolipoprotein E gene, play important roles in cardiovascular fitness and brain functioning in aging populations, so there is a critical need for future studies to examine the relationships among exercise modes, brain functioning, and such genetic variables.

## Conclusion

Our findings suggest that individuals with exercise experience, specifically those with open-skill and closed-skill exercise experience, exhibit improved WM and physical fitness, as determined by a spatial WM task and various fitness measurements, respectively, relative to those who have only engaged in irregular exercise. The fMRI results of this study clearly demonstrated that the open-skill exercise group exhibited greater neural activation in the prefrontal and ACC regions, implying a compensatory mechanism for coping with age-related neurodegradation of the brain. Moreover, the late middle-aged open-skill exercise group revealed greater neural activation in the hippocampus, suggesting a greater beneficial effect of open-skill exercise on hippocampus-related functioning for the elderly. Our findings support the notion that exercise promotes cognitive health and improves WM in late middle-aged adults and further suggest that various exercise modes effectively modulate frontal and hippocampal function, findings that may inform the selection of targeted activities for future exercise programs and interventions for the elderly.

## Data Availability

The raw data supporting the conclusions of this manuscript will be made available by the authors, without undue reservation, to any qualified researcher.

## Author Contributions

F-TC collected data and wrote the draft manuscript. Y-PC and C-MH interpreted and analyzed the data. SS and SC-K revised the manuscript. Y-KC, C-MH, and F-TC designed the study, analyzed and interpreted the data and revised the manuscript. Y-KC and C-MH supervised the project. All authors discussed the results and contributed to the final manuscript.

## Conflict of Interest Statement

The authors declare that the research was conducted in the absence of any commercial or financial relationships that could be construed as a potential conflict of interest.
